# Semaphorin3B Promotes Proliferation and Osteogenic Differentiation of Bone Marrow Mesenchymal Stem Cells in a High-Glucose Microenvironment

**DOI:** 10.1155/2021/6637176

**Published:** 2021-02-26

**Authors:** Quan Xing, Jingyi Feng, Xiaolei Zhang

**Affiliations:** ^1^Department of Zhujiang New Town Clinic, Guanghua School and Hospital of Stomatology, Sun Yat-sen University, Guangzhou 510055, China; ^2^Guangdong Provincial Key Laboratory of Stomatology, Sun Yat-sen University, Guangzhou, China; ^3^Department of Operative Dentistry and Endodontics, Guanghua School and Hospital of Stomatology, Sun Yat-sen University, Guangzhou 510055, China

## Abstract

Bone marrow mesenchymal stem cells (BMSCs) play an essential role in osteogenesis and bone metabolism and have already been recognized as one of the most popular seed cells for bone tissue engineering for bone diseases. However, high-glucose (HG) conditions in type 2 diabetes mellitus (T2DM) exert deleterious effects on BMSC proliferation and osteogenic differentiation. Semaphorin 3B (Sema3B) increases osteoblast differentiation in bone metabolism. Here, we determined the role of Sema3B in the proliferation and osteogenic differentiation of BMSCs in the HG microenvironment. The HG microenvironment decreased Sema3B expression in BMSCs. Moreover, HG inhibited BMSC proliferation. Furthermore, HG inhibited osteogenic differentiation in BMSCs by decreasing the expression of bone formation markers, alkaline phosphatase (ALP) activity, and mineralization. However, the administration of recombinant Sema3B reversed all of these effects. Moreover, our study found that Sema3B could activate the Akt pathway in BMSCs. Sema3B rescues defects in BMSC proliferation and osteogenic differentiation in the HG microenvironment by activating the Akt pathway. These effects were significantly reduced by treatment with an Akt inhibitor. Together, these findings demonstrate that Sema3B promotes the proliferation and osteogenic differentiation of BMSCs via the Akt pathway under HG conditions. Our study provides new insights into the potential ability of Sema3B to ameliorate BMSC proliferation and osteogenic differentiation in an HG microenvironment.

## 1. Introduction

Bone marrow mesenchymal stem cells (BMSCs) are characterized by differentiation into various types of cells, such as osteoblasts, chondrocytes, and other cell types [1–3]. BMSCs play a critical role in osteogenesis and bone metabolism [1–6]. Inhibition of the proliferation and osteogenic differentiation of BMSCs induces dysfunction of bone metabolism and gives rise to multiple bone loss diseases, including osteoporosis, periodontitis, and dental implantation failure [2, 7, 8]. Moreover, BMSCs have already become an attractive option as seed cells for the treatment of osteoblastic diseases [6–8]. However, many pathogenic factors suppress the proliferation and osteogenic differentiation of BMSCs, which leads to increased bone loss [9–12]. For instance, high-glucose conditions in type 2 diabetes mellitus (T2DM) exert deleterious effects on BMSC proliferation and osteogenic differentiation [9, 13–15].

T2DM is a highly prevalent metabolic disease globally and is characterized by excessive blood glucose, insulin resistance, and relative insulin deficiency [16]. T2DM is associated with impaired bone remodeling, osteopenia, osteoporosis, and other diabetes-related bone diseases [17–19]. Diabetes mellitus impairs bone metabolism, suppresses bone formation, and impedes fracture healing [20, 21]. Increasing evidence suggests that a high-glucose environment inhibits BMSC proliferation and osteogenic differentiation, inducing diabetic bone disease [9, 13, 15, 22–24]. Agents that could promote the osteogenic differentiation and proliferation of BMSCs may represent promising candidates to ameliorate the disorder of BMSCs in diabetic osteopathy.

As secreted glycoproteins on the cell surface, semaphorins are capable of regulating cell growth, cell differentiation, and cell migration in various tissues [25, 26]. A few studies showed that some semaphorin family proteins are crucial regulators of skeletal homeostasis [25–27]. Former studies have shown that semaphorin 3B (Sema3B) is associated with the bone metabolism process [28–30]. 1,25(OH)_2_D_3_ was suggested to increase transcription of Sema3B in osteoblasts [28]. Osteoblasts derived from transgenic mice expressing overexpression of Sema3B could promote osteogenic differentiation of BMSCs in vitro, whereas depletion of Sema3B impaired BMSC osteoblastic differentiation [29, 30]. Moreover, TNF-*α* decreased Sema3B expression by inhibiting Wnt signaling, but Sema3B overexpression reversed the osteogenic defects of BMSCs treated with TNF-*α* [29]. Wnt signaling is involved in this process [29]. However, the role of Sema3B in BMSC proliferation and osteogenic differentiation in a high-glucose microenvironment has still not been elucidated. Thus, the present study is aimed at investigating the effect of high glucose on the proliferation and osteogenic differentiation of BMSCs and clarifying the role of Sema3B in this process.

## 2. Materials and Methods

### 2.1. Cell Culture

Mouse BMSCs were isolated from six-week-old male C57BL/6 mice. Briefly, the femur and tibia bones were aseptically removed. The bone marrow was rinsed with culture medium consisting of *α*-MEM (Invitrogen, USA) supplement with 20% FBS (HyClone, Rockford) and 1% penicillin/streptomycin. Then, the cells were allowed to grow at 37°C with 5% CO_2_. BMSCs were separated by differential adhesion to culture plastic. The attached cells were grown in culture medium and used for further experiments. All procedures involving mice were approved by the Animal Ethical and Welfare Committee of Sun Yat-sen University, China.

In the high-glucose treatment (HG) group, BMSCs were subject to high glucose by incubation in a medium with 25 mM glucose. The normal-glucose treatment (NG)group was treated with 5.5 mM glucose. For inhibition of the Akt signaling pathway, cells were pretreated with an Akt inhibitor (MK2206) at a concentration of 10 *μ*M (Selleck, China).

### 2.2. Flow Cytometry

Marker expression on the BMSC surface was assessed using FACSCalibur (BD Biosciences, USA). Briefly, BMSCs at passage 3 were trypsinized, washed, and resuspended in PBS. Then, 2 × 10^5^ cells were incubated with PE- or FITC-labeled mouse cell surface marker antibodies against CD29, CD44, CD34, and CD45 (BD Biosciences), as well as isotype control antibodies at 4°C for 30 min. Then, the samples were analyzed using FACSCalibur.

### 2.3. Real-Time PCR

Total RNA was isolated, and reverse transcription was performed using an Omniscript Reverse Transcription Kit (Qiagen, Germany). The primers used for the target sequence are listed in Table 1. Real-time PCR was performed by the QuantiTect SYBR® Green PCR Kit (Qiagen). The relative mRNA expression levels of the target genes were quantified in comparison to the expression of *β*-actin using the 2^-*ΔΔ*^CT method. Real-time PCR results are presented as the mean ± SEM.

### 2.4. Western Blotting

Total protein was prepared with M-PER Protein Extraction Reagent (Thermo Scientific). Protein concentrations were measured using a BCA protein assay reagent (Pierce). Briefly, 30 mg of sample was subjected to 10% SDS-PAGE and electrotransferred onto a PVDF membrane. The following primary antibodies were used for western blotting : Sema3B (1 : 1000, Abcam, Cambridge, UK), phospho-Akt (Ser473) (p-Akt) (1 : 1000, Cell Signaling Technology, USA), Akt (1 : 1000, Cell Signaling Technology), and *β*-actin (1 : 4000) (Santa Cruz Biotechnology, USA). PVDF membranes were then incubated with an appropriate horseradish peroxide-conjugated antibody (Santa Cruz Biotechnology). The signal was visualized using a chemiluminescent reagent kit (Pierce). Relative band densities were calculated (Image J 1.8.0, NIH).

### 2.5. ALP Activity

BMSCs (1 × 10^4^ cells/cm^2^) were treated with 100 ng/ml Sema3B (R&D Systems, USA) in NG or HG medium for up to 14 days. Total protein was extracted from cells on ice at days 7 and 14 using M-PER Protein Extraction Reagent (Thermo Scientific). Protein concentrations were assessed using a BCA protein assay reagent (Pierce). ALP activity was detected using an ALP activity detection kit (Sigma-Aldrich).

### 2.6. Mineralization Assay

BMSCs were treated with recombinant 100 ng/ml Sema3B in osteogenic differentiation media with NG or HG for 14 days. The culture medium was renewed every three days. Mineralization was identified by alizarin red staining on the 14th day as previously described [31]. Then, the cells were treated with ethylpyridium chloride and quantified at 550 nm.

### 2.7. Osteogenic and Adipogenic Differentiation of BMSCs

For adipogenesis, BMSCs (1 × 10^4^ cells/cm^2^) were cultured in adipogenic differentiation medium containing with 0.1 *μ*M dexamethasone,1 *μ*M insulin, 200 *μ*M domethacin, and 250 *μ* Misobutylmethylxanthine (Sigma-Aldrich). The medium was replaced every 3 days. Twenty-one days later, the cells were fixed with formalin and subjected to 0.5% (*w*/*v*) oil red O staining (Sigma-Aldrich).

For osteogenic differentiation, BMSCs (1 × 10^4^ cells/cm^2^) were cultured in osteogenic differentiation medium containing *α*-MEM containing 10% FBS, 10 mM *β*-glycerophosphate, and 50 mg/ml ascorbic acid (Sigma-Aldrich). Twenty-one days later, the cells were fixed and stained with alizarin red (Sigma-Aldrich).

### 2.8. Cell Proliferation Assay

Briefly, BMSCs were seeded in and incubated with experimental compounds for 24, 48, and 72 h. Then, 10 *μ*l of CCK-8 solution (Dojindo Molecular Technologies, Japan) was added to each well at the end of the experiment. The absorbance was measured at 450 nm 2 h later.

### 2.9. Statistical Analysis

All data are shown as the mean ± SEM. Statistical comparisons were made by one-way ANOVA or two-way ANOVA as appropriate. A value of *P* < 0.05 was considered statistically significant.

## 3. Results

### 3.1. Characterization of Mouse BMSCs

**Figure 1:**
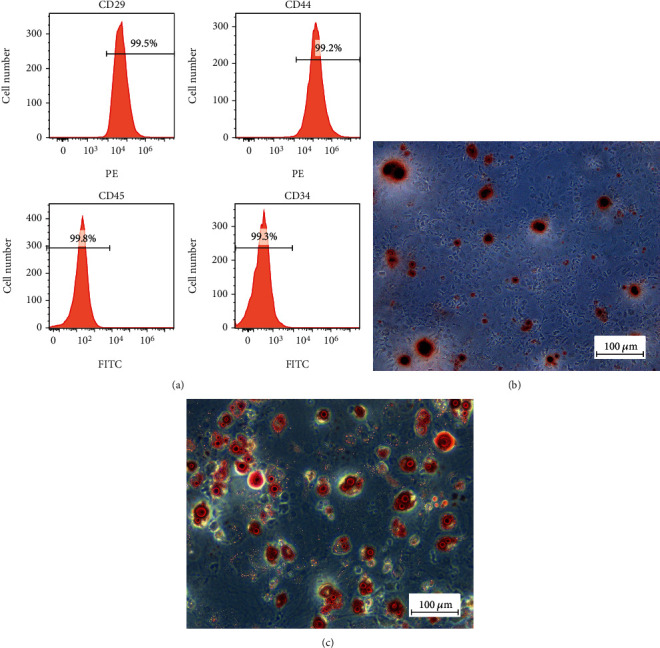
Identification of BMSCs. Cells were evaluated by surface markers and their multipotency to differentiate. (a) Cells were stained with FITC- or PE-conjugated antibodies. The differentiation of BMSCs using an induction medium induces cell differentiation into osteocytes and adipocytes. The multipotency of BMSCs was confirmed as positive by alizarin red (b) and oil red O staining (c), indicating the differentiation of BMSCs into osteocytes and adipocytes, respectively.

The phenotypic characteristics of BMSCs were analyzed by flow cytometry, demonstrating that the cells were CD29+, CD44+, CD45-, and CD34-. The results are shown in Figure 1(a).

As pluripotent stem cells, BMSCs were assessed for their ability to differentiate into osteoblasts using alizarin red and adipocytes using oil red O staining. The results showed positive staining, as demonstrated in Figures 1(b) and 1(c).

### 3.2. HG Suppressed Sema3B Expression in BMSCs

**Figure 2:**
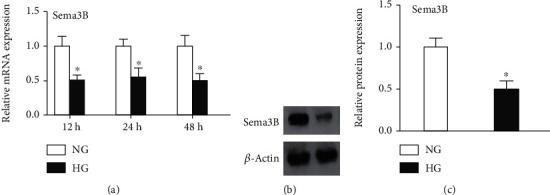
Effects of HG onSema3B expression. Sema3B expression levels were measured by real-time PCR and western blotting. (a) HG treatment suppressed Sema3B mRNA expression in BMSCs at 24, 48, and 72 h. (b) Representative western blots. (c) HG decreased Sema3B protein expression in BMSCs. Data are expressed as the mean ± SEM (*n* = 3). ^∗^*P* < 0.05 compared to the control sample.

To assess the effects of HG on Sema3B expression, the mRNA and protein levels of Sema3B in BMSCs were measured by real-time PCR and western blotting. Real-time PCR demonstrated that HG significantly decreased Sema3B mRNA levels in BMSCs (*P* < 0.05) (Figure 2(a)). Western blotting revealed that HG significantly reduced Sema3B protein expression (*P* < 0.05) (Figures 2(b) and 2(c)).

### 3.3. The Effect of Sema3B on BMSC Proliferation under HG Conditions

**Figure 3:**
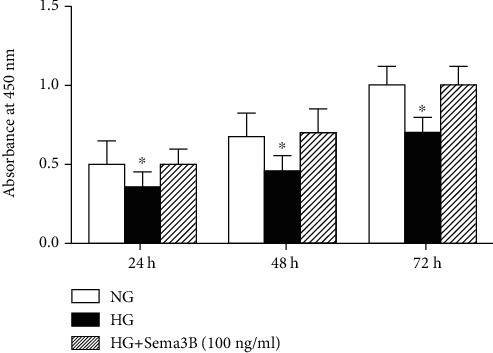
Effects of Sema3B on BMSC proliferation under HG treatment. The proliferation of BMSCs cultured under HG conditions was analyzed by CCK-8 assays at 24, 48, and 72 h. The results are represented the mean ± SEM (*n* = 3). ^∗^*P* < 0.05 compared to the other sample.

To detect the effects of Sema3B on BMSC proliferation, we measured BMSC proliferation under HG conditions using CCK-8 assays. HG significantly decreased BMSC proliferation compared with that of any of the other groups at each indicated time point. Moreover, recombinant Sema3B reversed the reduction in cell proliferation (*P* < 0.05) (Figure 3(a)).

### 3.4. Sema3B Alleviates the Inhibition of Osteogenic Differentiation Markers

**Figure 4:**
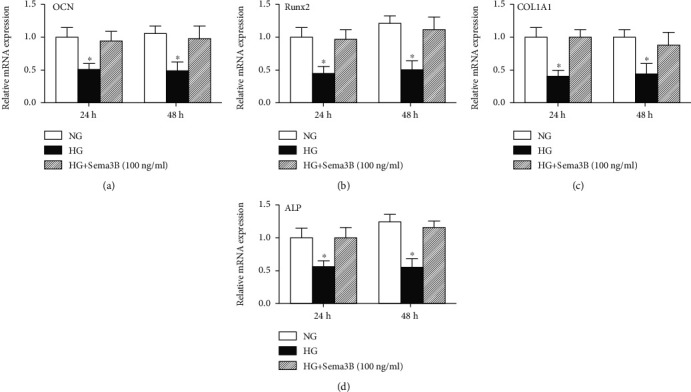
Effects of Sema3B on osteogenic markers under HG treatment. OCN (a), Runx2 (b), COL1A1 (c), and ALP (d) mRNA expression levels in BMSCs were measured by real-time PCR at each time point. Real-time PCR results are expressed as the mean ± SEM (*n* = 3). ^∗^*P* < 0.05 compared to the other samples.

To investigate the effects of Sema3B on BMSCs in the HG microenvironment, we assessed the expression of specific osteogenesis markers by real-time PCR. The results showed that osteocalcin (OCN), Runx2, type I collagen*α*1 (COL1A1), and ALP mRNA levels in BMSCs were markedly decreased upon treatment with HG. Furthermore, Sema3B markedly rescued the decrease in OCN, Runx2, COL1A1, and ALP mRNA expression levels in the HG groups (Figures 4(a)–4(d)).

### 3.5. Sema3B Ameliorates HG-Induced Inhibition of Osteogenic Differentiation

To investigate the role of Sema3B in osteogenic differentiation, we measured ALP activity and mineralization in BMSCs with and without Sema3B (100 ng/ml) treatment.

**Figure 5:**
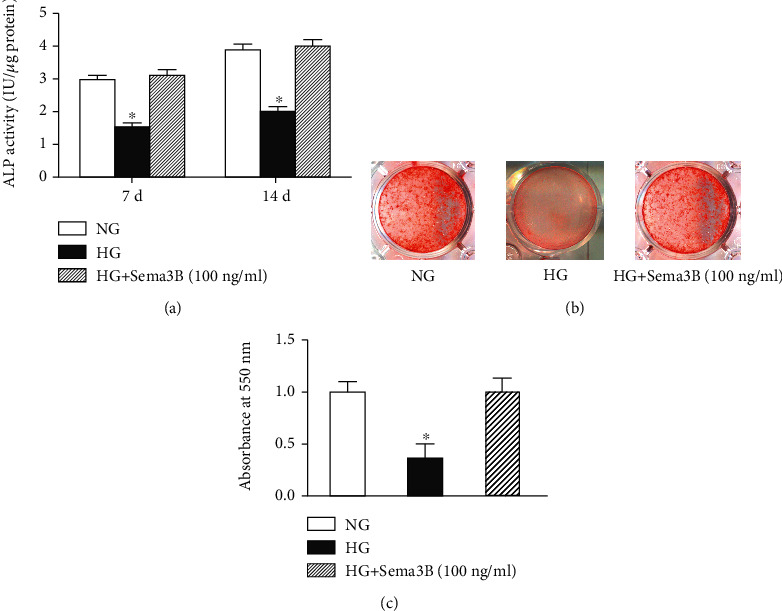
Effects of Sema3B on osteogenic differentiation under HG treatment. (a) ALP activity is presented as the mean ± SEM (*n* = 3). (b) Alizarin red S staining. (c) Quantification of alizarin red staining. The results of alizarin red staining are presented as the mean ± SEM (*n* = 3). ^∗^*P* < 0.05 compared to the other samples.

HG significantly reduced ALP activity in BMSCs at 7 days (1.50 IU/*μ*g) and 14 days (2.01 IU/*μ*g) (*P* < 0.05). Sema3B administration significantly reversed the decrease in ALP activity at the indicated time points (3.10 IU/*μ*g at 7 days and 4.05 IU/*μ*g at 14 days) (*P* < 0.05) (Figure 5(a)).

Mineralization was significantly decreased by HG treatment. The administration of Sema3B significantly rescued the HG-induced inhibition of mineralization in BMSCs (*P* < 0.05) (Figures 5(b) and 5(c)).

### 3.6. Sema3B Rescues HG-Induced Inhibition of the Akt Pathway

**Figure 6:**
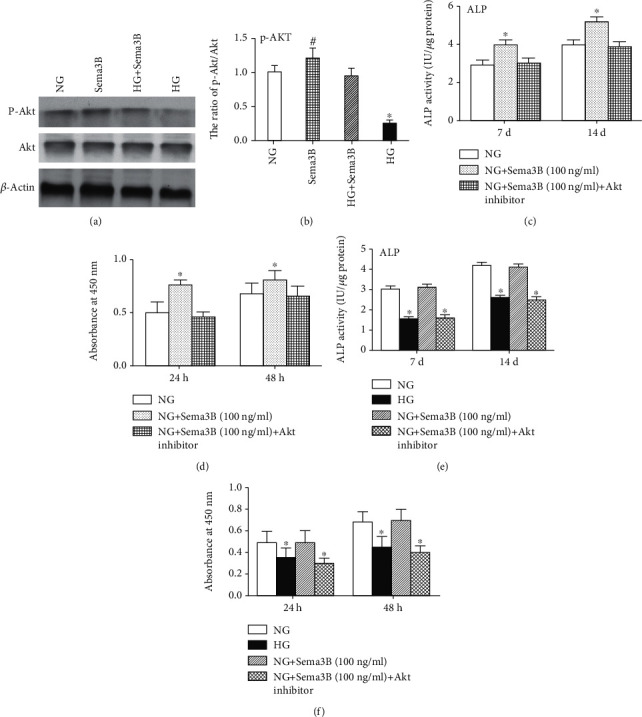
Effects of Sema3B on BMSCs by the Akt pathway under HG treatment. (a) Representative western blots are shown. (b) The expression levels of phospho-Akt and Akt were evaluated by western blotting. (c) ALP activity of BMSCs was detected using an ALP activity detection kit. (d) BMSC proliferation was assessed by CCK-8 assay. Cells were pretreated with an Akt inhibitor (MK2206) (10 *μ*M) for 2 h followed by stimulation with HG or HG + Sema3B. Then, (e) ALP activity of BMSCs and (f) the proliferation of BMSCs were assessed. Data are expressed as the mean ± SEM (*n* = 3). ^∗^*P* < 0.05 compared to the other samples, ^#^*P* < 0.05 compared to the control sample.

In this experiment, we investigated the relationship between Akt signaling and Sema3B in BMSCs proliferation and osteogenic differentiation under an HG microenvironment. HG significantly decreased Akt phosphorylation compared with the NG group. However, treatment of cells with Sema3B recovered the reduced levels of Akt phosphorylation observed under HG conditions (*P* < 0.05) (Figures 6(a) and 6(b)).

Moreover, Sema3B elevated ALP activity and proliferation in BMSCs. Inhibition of the Akt pathway abolished the Sema3B-mediated induction of ALP activity and proliferation in BMSCs (*P* < 0.05) (Figures 6(c) and 6(d)).

Furthermore, BMSCs are incubated with MK2206 prior to Sema3B administration. The results demonstrated that Sema3B restored HG-mediated suppression of ALP activity and proliferation. However, the protective effect of Sema3B was inhibited by pretreatment with an Akt inhibitor (*P* < 0.05) (Figures 6(e) and 6(f)). The results indicated that Sema3B ameliorates the suppression of BMSC proliferation and osteogenesis under an HG microenvironment via the Akt pathway.

## 4. Discussion

BMSCs derived from bone marrow stroma or connective tissue are easy to isolate and undergo pluripotent differentiation; thus, these cells are considered useful for applications in a variety of clinical therapies [8, 32–35]. In recent years, accumulating studies have demonstrated that BMSCs can be used in bone defect repair due to their capacity for osteogenic differentiation [3, 4, 6, 8]. However, many pathologic factors decrease proliferation and osteogenic differentiation, such as inflammation, a high-glucose environment, and other factors [9, 11–13]. Hyperglycemia is a major cause of a series of diabetic complications, including diabetic osteopathy [15, 20]. High glucose has been reported to inhibit the proliferation and osteogenic differentiation of BMSCs through the BMP signaling pathway [36]. Moreover, optimal glycemic control has been recognized to associate with successfully osseointegrated dental implantation in diabetic patients [37]. In our study, HG decreased BMSC proliferation. Moreover, HG reduced the expression of osteogenesis markers and osteogenic differentiation in BMSCs. The results were consistent with previous studies. However, the osteogenic differentiation of BMSCs under HG conditions is not completely understood. Identifying factors that could promote BMSC proliferation and osteogenic differentiation in the HG microenvironment may reveal promising candidates that can ameliorate the function of BMSCs in hyperglycemia-induced bone diseases.

The involvement of Sema3B in bone metabolism has been reported in previous studies [28–30]. Sema3B was confirmed to enhance osteogenic differentiation of BMSCs [29, 30]. Moreover, Sema3B was found to be involved in estrogen deficiency-induced osteoporosis [29]. The effect of HG on BMSC proliferation was analyzed by CCK-8 assay. Based on these results, HG caused significant inhibition of BMSC proliferation compared with that of the NG group. In addition, Sema3B treatment markedly reversed HG-induced inhibition of BMSC proliferation.

Our data also demonstrated that Sema3B reversed the HG-induced decrease in OCN, Runx2, ALP, and COL1A1 mRNA expression in BMSCs. Furthermore, Sema3B attenuated HG-induced reduction of ALP activity and mineralization in BMSCs. Therefore, these observations may suggest that Sema3B is a promising candidate for promoting osteogenesis of BMSCs in an HG microenvironment.

The Akt pathway plays an essential role in bone development and skeletal remodeling [38, 39]. Recently, numerous studies have shown that high glucose suppresses proliferation and osteogenic differentiation by inactivating the PI3K/Akt signaling pathway in osteoblasts [22, 40, 41]. In this present experiment, we observed that Sema3B significantly increased Akt phosphorylation. However, pretreatment with an Akt inhibitor eliminated the increased proliferation and ALP activity of BMSCs in the HG microenvironment, suggesting that Sema3B promotes osteogenic differentiation of BMSCs under HG conditions by modulating the Akt pathway.

Thus, we found that HG impaired the proliferation and osteogenic differentiation of BMSCs. Furthermore, our study demonstrated that Sema3B could prevent HG-mediated BMSC dysfunction through modulation of the Akt pathway. Sema3B might represent a promising agent to ameliorate the proliferation and osteogenic differentiation of BMSCs in an HG microenvironment. Therefore, our study may lead to new insights into more effective clinical interventions for hyperglycemia-related bone diseases.

## Figures and Tables

**Table 1:** Primer sequence used for real-time PCR analysis.

Gene forward primer sequence (5′-3′)/reverse primer sequence (5′-3′)	
Sema3B [29]	CTTCGGCTCTCCTTTCAAGA
	CAAGGCTTCATAACAGCAGGT
OCN [43]	CTGCGCTCTGTCTCTCTGAC
	TTAAGCTCACACTGCTCCCG
ALP [43]	CCGGCTGGAGATGGACAAAT
	CTCATTGCCCTGAGTGGTG
Runx2 [43]	AAATGCCTCCGCTGTTATGAA
	GCTCCGGCCCACAAATCT
COL1A1 [44]	GCAACAGTCGCTTCACCTACA
	CAATGTCCAAGGGAGCCACAT
*β*-Actin [42]	TGACAGGATGCAGAAGGAGA
	CGCTCAGGAGGAGCAATG

## Data Availability

The data used to support the findings of this study are available from the corresponding author upon reasonable request.
